# Impact on antimicrobial consumption of procalcitonin-guided antibiotic therapy for pneumonia/pneumonitis associated with aspiration in comatose mechanically ventilated patients: a multicenter, randomized controlled study

**DOI:** 10.1186/s13613-021-00931-4

**Published:** 2021-10-12

**Authors:** Guylaine Labro, François Aptel, Marc Puyraveau, Jonathan Paillot, Sébastien Pili Floury, Hamid Merdji, Julie Helms, Gaël Piton, Fiona Ecarnot, Khaldoun Kuteifan, Jean Pierre Quenot, Gilles Capellier, for the PROPASPI (PROcalcitonin Pneumonia/pneumonitis Associated with ASPIration) trial investigators, Jean-Christophe Navellou, Claire Chaignat, Mathilde Grandperrin, Mélanie Claveau, Nicolas Belin, Cyrille Patry, Frédéric Claude, François Belon, Loïc Barrot, Marion Colnot, Guillaume Besch, Gilles Blasco, Marc Ginet, Yannick Brunin, Pascal Andreu, Auguste Dargent, Pierre Emmanuel Charles, Ferhat Meziani, Alexandra Monnier, Antoine Studer, Raphaël Clere-Jehl, Hassene Rahmani, Anne Florence Dureau, Antoine Poidevin, Joy Mootien, Gokhan Bodur, Carmen Ionescu, Philippe Guiot

**Affiliations:** 1 Medical Intensive Care Unit, University Hospital, Besançon, France; 2 Medical Intensive Care Unit, Hospital of Mulhouse, Mulhouse, France; 3 Department of Intensive Care, François Mitterrand University Hospital, Dijon, France; 4 INSERM Clinical Investigation Center 1431, Besançon, France; 5 Surgical Intensive Care Unit, University Hospital, Besançon, France; 6 EA3920, University of Burgundy Franche-Comté, 25000 Besancon, France; 7 Service de Médecine Intensive Réanimation, Nouvel Hôpital Civil, Université de Strasbourg, Hôpitaux Universitaires de Strasbourg, Strasbourg, France; 8 UMR 1260, Fédération de Médecine Translationnelle de Strasbourg (FMTS), INSERM, Strasbourg, France; 9 Department of Cardiology, University Hospital, Besançon, France; 10 Lipness Team, INSERM Research Center LNC-UMR1231 and LabEx LipSTIC, University of Burgundy, Dijon, France; 11 INSERM CIC 1432, Clinical Epidemiology, University of Burgundy, Dijon, France; 12 Australian and New Zealand Intensive Care Research Centre, Department of Epidemiology and Preventive Medicine, Monash University, Melbourne, Australia

**Keywords:** Aspiration, Pneumonia, Procalcitonin, Intensive care unit, Coma

## Abstract

**Background:**

In comatose patients receiving oro-tracheal intubation for mechanical ventilation (MV), the risk of aspiration is increased. Aspiration can lead to chemical pneumonitis (inflammatory reaction to the gastric contents), or aspiration pneumonia (infection caused by inhalation of microorganisms). Distinguishing between the two types is challenging. We tested the interest of using a decisional algorithm based on procalcitonin (PCT) values to guide initiation and discontinuation of antibiotic therapies in intubated patients.

**Methods:**

The PROPASPI (PROcalcitonin Pneumonia/pneumonitis Associated with ASPIration) trial is a multicenter, prospective, randomized, controlled, single-blind, superiority study comparing two strategies: (1) an intervention group where threshold PCT values were used to guide initiation and discontinuation of antibiotics (PCT group); and (2) a control group, where antibiotic therapy was managed at the physician’s discretion. Patients aged 18 years or over, intubated for coma (Glasgow score ≤ 8), with MV initiated within 48 h after admission, were eligible. The primary endpoint was the duration of antibiotic treatment during the first 15 days after admission to the ICU.

**Results:**

From 24/2/2015 to 28/8/2019, 1712 patients were intubated for coma in the 5 participating centers, of whom 166 were included in the study. Data from 159 were available for intention-to-treat analysis: 81 in the PCT group, and 78 in the control group. Overall, 67 patients (43%) received antibiotics in the intensive care unit (ICU); there was no significant difference between groups (37 (46%) vs 30 (40%) for PCT vs control, *p* = 0.432). The mean duration of antibiotic treatment during the first 15 days in the ICU was 2.7 ± 3.8 days; there was no significant difference between groups (3.0 ± 4.1 days vs 2.3 ± 3.4 days for PCT vs control, *p* = 0.311). The mean number of days under MV was significantly higher in the PCT group (3.7 ± 3.6 days) than in controls (2.7 ± 2.5 days, *p* = 0.033). The duration of ICU stay was also significantly longer in the PCT group: 6.4 ± 6.5 days vs 4.6 ± 3.5 days in the control group (*p* = 0.043). After adjustment for SAPS II score, the difference in length of stay and duration of mechanical ventilation between groups was no longer significant.

**Conclusion:**

The use of PCT values to guide therapy, in comparison to the use of clinical, biological (apart from PCT) and radiological criteria, does not modify exposure to antibiotics in patients intubated for coma.

*Trial registration* Clinicaltrials.gov Identifier NCT02862314.

**Supplementary Information:**

The online version contains supplementary material available at 10.1186/s13613-021-00931-4.

## Introduction

Inhalation pneumonia is defined as inhalation of stomach content or oropharyngeal secretions into the larynx and lower respiratory tract [1]. Consciousness disorders [2] and intubation performed in emergency circumstances are among the main risk factors for inhalation [3, 4]. Two types of inhalation pneumonia can be distinguished, namely bacterial and chemical. Aspiration pneumonia is infection caused by inhalation of microorganisms, whereas chemical pneumonitis is an inflammatory reaction to the gastric contents [5]. The distinction between the two can be complex, due to clinical and radiological similarities [6]. Consequently, many patients with suspected inhalation receive antibiotic therapy without bacterial infection being confirmed [7–9].

Some authors have proposed starting systemic antibiotic treatment in all patients who required intubation for coma [10–14]. A meta-analysis by Righy et al. [15] showed that a strategy of systematic antibiotic treatment in patients requiring intubation for coma was associated with a significantly decreased incidence of early-onset ventilator-acquired pneumonia (VAP), and with a shorter length of ICU stay. However, this meta-analysis did not show any effect on mortality or duration of MV.

Prophylactic use of antibiotic therapy after oro-tracheal intubation for patients intubated for coma is not currently recommended [16, 17]. When inhalation pneumonia is suspected, early initiation of antibiotics must be weighed against the risks inherent to antibiotic use, such as *Clostridium difficile* infections [18], or the emergence of multi-drug resistant bacteria [19].

Procalcitonin (PCT) is among the most widely used and best known biomarkers for the detection of bacterial infection in the ICU setting [20]. With a view to minimizing unnecessary antibiotic use, several studies have investigated PCT-guided antibiotic treatment, especially in the ICU [21–24]. A recent meta-analysis of randomized clinical trials testing the clinical impact of procalcitonin-based algorithms for the duration of antibiotic treatment in critically ill adult patients with sepsis or septic shock [25] found that the PCT-guided strategy was associated with a significantly shorter duration of antibiotic therapy, and a significant decrease in mortality. Other observational studies [26–29] have evaluated whether PCT could be used to distinguish between bacterial and chemical aspiration pneumonia in patients receiving invasive MV, with conflicting results.

In this context, this prospective, randomized, interventional study aimed to investigate the impact of using PCT dosage to guide antibiotic treatment in patients intubated for a coma on the duration of exposure to antibiotics.

## Material and methods

### Settings and design

#### Study design

The PROPASPI study was a randomized, prospective, multicenter, controlled, open-label superiority trial with parallel groups, performed in 5 ICUs (4 medical and 1 surgical) in 3 university hospitals and one general (non-academic) hospital in France. The study was performed in accordance with the Declaration of Helsinki and the tenets of Good Clinical Practice. The study received central approval for all study sites from the Ethics Committee Comité de Protection des Personnes (CPP) Est-II in April 2014 under the number 14/427, and from the French Agency for the Safety of Medications and Health Products (Agence Nationale de Sécurité du Médicament et des produits de santé, ANSM) under the number 2013-A01802-43*.* Since included patients were in a coma at the time of inclusion, their legal representative was informed about the study, and provided written informed consent. Patients were informed of their inclusion in the study and their written consent was obtained as soon as they regained capacity.

#### Participants

Patients were eligible for inclusion in the study if they were aged 18 or older, had undergone oro-tracheal intubation for a coma (Glasgow Coma Score ≤ 8), with MV initiated in the first 48 h after hospital admission.

Exclusion criteria were pregnancy, patients aged < 18 years, patients under legal protection, patients without health insurance, patients included in another interventional clinical study involving infections or antibiotics or having the same primary outcome, moribund patients, patients with conditions in which PCT concentrations could be increased without any relation to an infectious process (e.g., trauma patients, surgery within the last 4 days, cardiorespiratory arrest, administration of anti-thymocyte globulin), immunosuppression (bone marrow transplant, patients with severe neutropenia), patients with an absolute indication for administration of antibiotics at the time of ICU admission (meningitis, pneumonia) or a chronic infection requiring prolonged antibiotic treatment (endocarditis, osteo-articular infections, mediastinitis, deep abscess, pneumocystis infection, toxoplasmosis, tuberculosis) and patients with hemodynamic instability of septic origin or respiratory insufficiency (defined as a PaO_2_/FiO_2_ ratio ≤ 200 mmHg and PEP ≥ 5 cmH_2_O).

### Study procedures and assessments

#### Randomization

Randomization was performed using dedicated software (CleanWeb™). Patients were randomly allocated to the interventional or control group in a 1:1 ratio. Randomization was performed in blocks of four and stratified by center.

### Procedures

#### PCT group

**Fig. 1:**
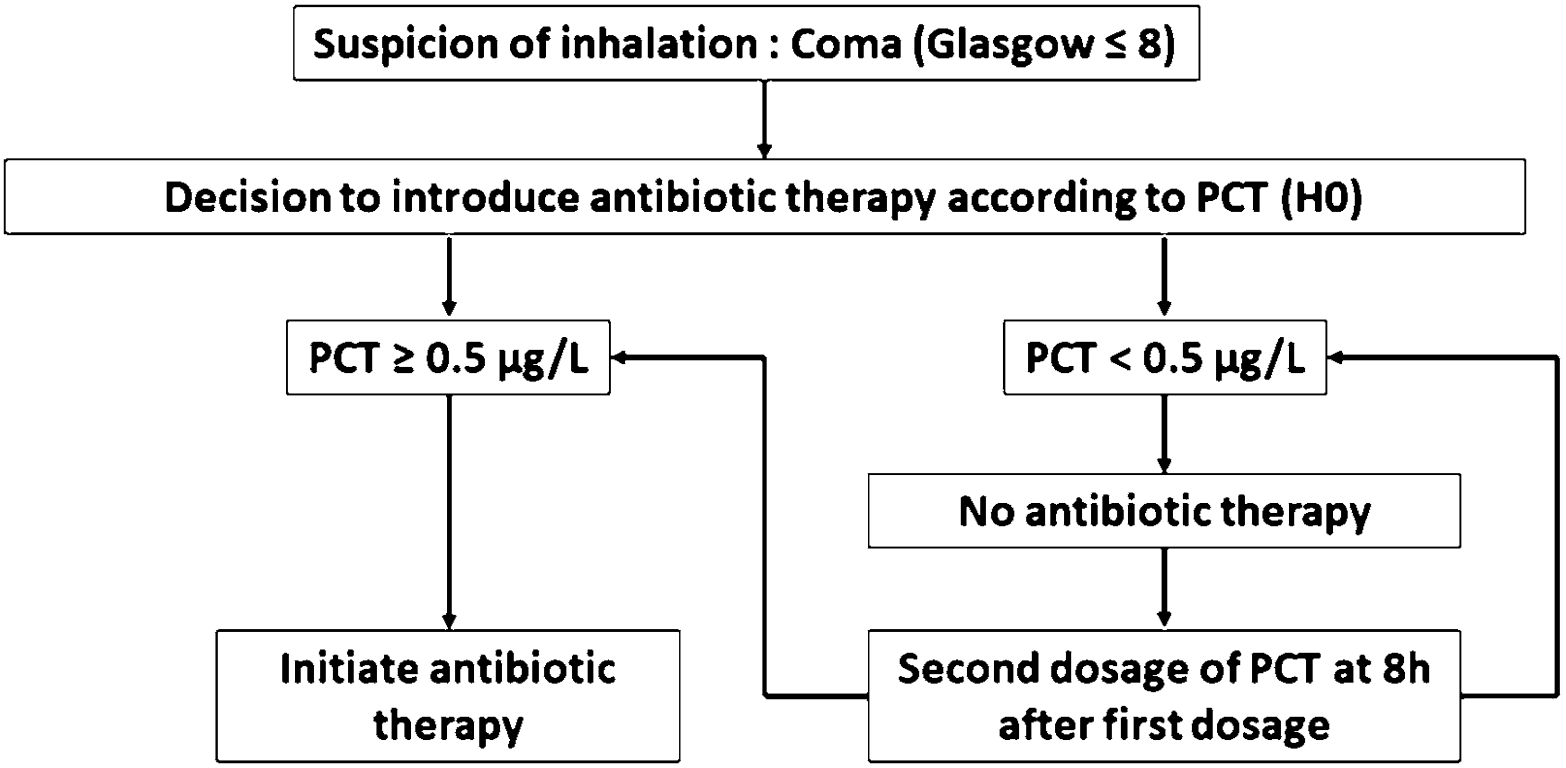
Decisional algorithm for the initiation of antibiotic therapy at inclusion (H0) and at 8 h after inclusion (H8)

In the PCT group, PCT concentration was measured at inclusion. If PCT concentration was ≥ 0.5 ng/mL, initiation of antibiotic treatment was recommended, and if PCT concentration was < 0.5 ng/mL, blood sampling was repeated after 8 h, and then on the second and third day after inclusion. If the PCT concentration rose to ≥ 0.5 ng/mL, initiation of antibiotic treatment was recommended. The decisional algorithm for the introduction of antibiotics is detailed in Fig. 1.

**Fig. 2:**
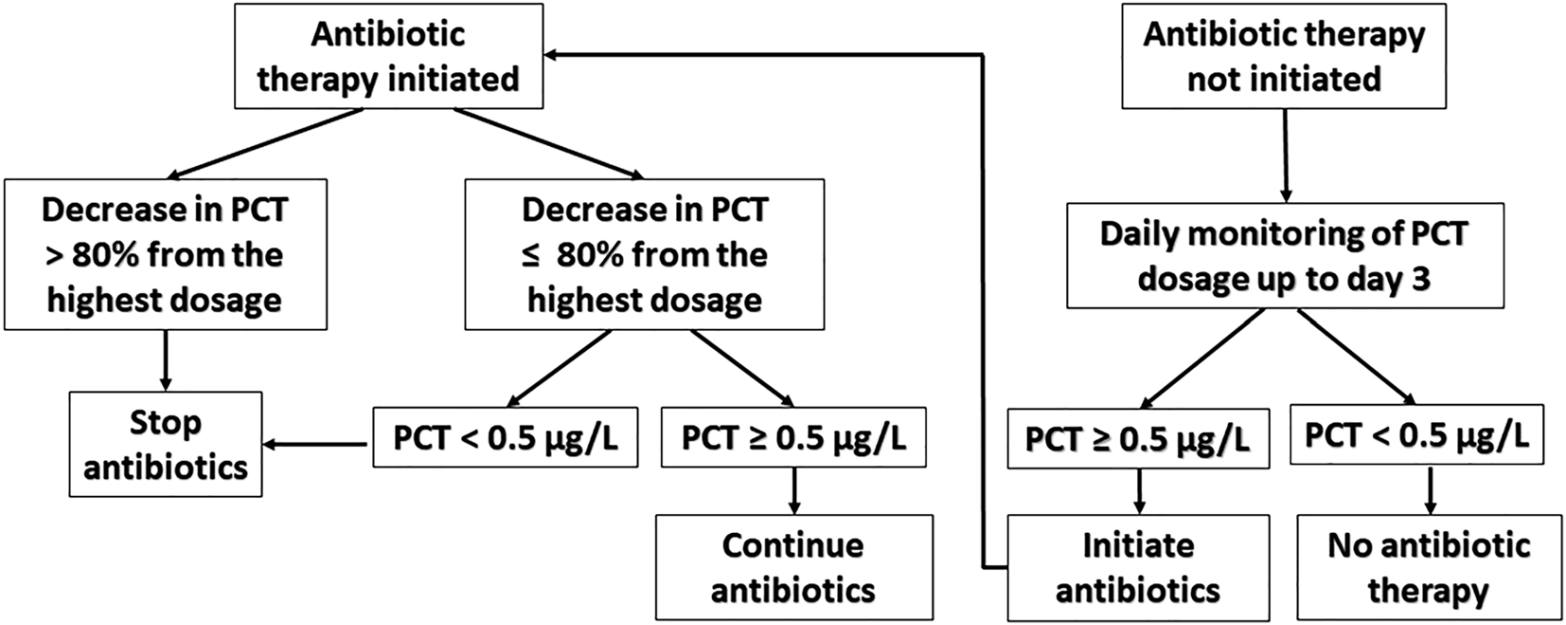
Decisional algorithm for the discontinuation of antibiotic therapy and patient follow-up

Once antibiotic treatment was initiated, PCT concentrations were monitored daily until discontinuation of antibiotic treatment or discharge from the ICU. If the PCT concentration declined by more than 80% of its maximal value or dropped below 0.5 ng/mL, discontinuation of antibiotic treatment was recommended. The decisional algorithm for the discontinuation of antibiotics is detailed in Fig. 2.

The quantitative determination of procalcitonin was performed with TRACE (Time Resolved Amplified Cryptate Emission). Procalcitonin was measured with the automated B.R.A.H.M.S. Kryptor platform (Thermo Fisher Scientific, Hennigsdorf, Germany) and ADVIA Centaur BRAHMS® (Siemens Healthcare Diagnostics).

If infection caused by *Pseudomonas aeruginosa*, *Acinetobacter baumannii* or *Legionella pneumophila*, or infection requiring long-term antibiotic treatment was documented, the duration of antibiotic therapy was at the discretion of the physician in charge according to recommendations and local practice. If the patient was hemodynamically unstable due to septic shock or respiratory insufficiency (defined as a PaO_2_/FiO_2_ ratio ≤ 200 mmHg and PEP ≥ 5 cmH_2_O), it was recommended to introduce or continue antibiotic treatment.

The decisional algorithms were provided as guidance for antibiotic treatment, but the final decision regarding initiation and discontinuation of antibiotic treatment was left at the discretion of the treating physician. PCT was the only criterion for the introduction of antibiotics in the PCT group, unless there were signs of clinical severity representing a major risk for the patient (namely, septic shock or hypoxemia). The algorithm for introduction of antibiotics was supposed to be applied for the first 3 days only after inclusion in the study. Beyond day 3 after randomization, PCT was no longer measured, and was therefore no longer taken into account in the decision to introduce antibiotics. If the treating physician did not follow the recommendations in the algorithm, we recorded the reasons for the divergence.

#### Control group

In the control group, PCT concentrations were not measured. Initiation and discontinuation of antibiotic treatment was at the discretion of the treating physician. Antibiotics were started when there was a high clinical suspicion of aspiration pneumonia, which was to be suspected in the presence of the following signs and symptoms: clinical signs (hyperthermia > 38 °C without other plausible cause or hypothermia < 36 °C, appearance of purulent secretions, hemodynamic deterioration and/or respiratory deterioration without other plausible cause), biological signs [hyperleukocytosis > 12 G/L leucocytes, or leukopenia 4 G/L leukocytes, or elevation of inflammatory markers (other than PCT)], radiological signs (appearance, change or presence of radiological abnormalities compatible with pneumonia) [30]. Determination of other inflammatory markers, such as C-reactive protein (CRP), was at the discretion of the treating physician. Once antibiotic therapy was initiated, treatment was to be adapted according to the results of bacteriological cultures. The maximum duration of antibiotic therapy recommended was 8 days in the presence of favorable outcome. If lung infection was found to be caused by *Pseudomonas aeruginosa*, the recommended duration of antibiotic therapy was 15 days.

In both groups, additional investigations were performed at the time of inclusion and before the administration of antibiotics, namely: microbiological culture of tracheal aspirations, two sets of blood cultures (aerobic and anaerobic cultures), urine culture. When pneumonia was suspected, culture of tracheal aspirations was performed, and broncho-alveolar lavage was recommended in case of nosocomial pneumonia. Other cultures could be performed depending on the clinical presentation (skin biopsy, lumbar puncture, joint puncture, etc.). The emergence of multiresistant strains was detected twice a week in tracheal aspirations and once a week by rectal swab until discharge from the ICU.

When antibiotic therapy was initiated in case of inhalation pneumonia, treatment had to start with a molecule covering anaerobic bacteria. If antibiotic treatment was initiated for infection of a site other than the lungs, the duration of therapy had to follow national or international guidelines specific to the focus of infection.

The algorithm for discontinuation of antibiotic therapy was not applied after discharge from the ICU to another hospital ward, and therefore, adherence to this algorithm was not assessed.

### Definitions

Septic shock and severe sepsis were defined according to the Surviving Sepsis Campaign [31]. Duration of antibiotic therapy was calculated in terms of Defined Daily Doses (DDD) available from the WHO at http://www.whocc.no/atcddd/indexdatabase/.

### Outcomes

The primary endpoint was the duration of antibiotic treatment during the first 15 days after admission to the ICU.

### Secondary endpoints

The secondary endpoints were the duration of antibiotic treatment during the first 7 days following admission to the ICU; the cost of antibiotic treatment (calculated in DDD); the proportion of early pneumonia (occurring within 7 days of intubation), the occurrence of infection relapse, superinfections or infectious respiratory complications (lung abscess or purulent pleuritis) up to 28 days following admission to the ICU; duration of mechanical ventilation; duration of hospitalization in the ICU and in-hospital; disease severity, as assessed by the Sepsis-related Organ Failure Assessment score (SOFA) score at days 1, 3, 5, 7 and 15 after admission; mortality at 28 and 90 days.

### Sample size calculation

The sample size for the study was calculated based on the primary objective of the study, i.e., the number of days of antibiotic treatment in the first 15 days of hospitalization in the ICU, based on the following assumptions, and using nQuery Advisor software: estimated duration of the antibiotic treatment of 6.2 ± 3.3 days [32], an alpha risk of 5%, power of 90%, a reduction of 25% in the duration of antibiotic treatment in the intervention arm based on PCT concentrations, in a one-sided situation. Under these assumptions, the number of patients to be included was 74 per group. With an estimated 10% attrition, the total number to be included was 164 patients.

### Statistical analysis

Statistical analysis was performed according to the intention-to-treat principle. Quantitative data are described as mean ± standard deviation (SD) and qualitative variables as number (percentage). Groups were compared using the Student *t* or Mann–Whitney test for quantitative variables, and the chi square or Fisher’s exact test for qualitative variables, as appropriate. For analysis of the primary endpoint (duration of antibiotic therapy), groups were compared using the Student *t* test. Data collection for the study was performed using CleanWeb™ software (Telemedicine Technologies).

## Results

**Fig. 3:**
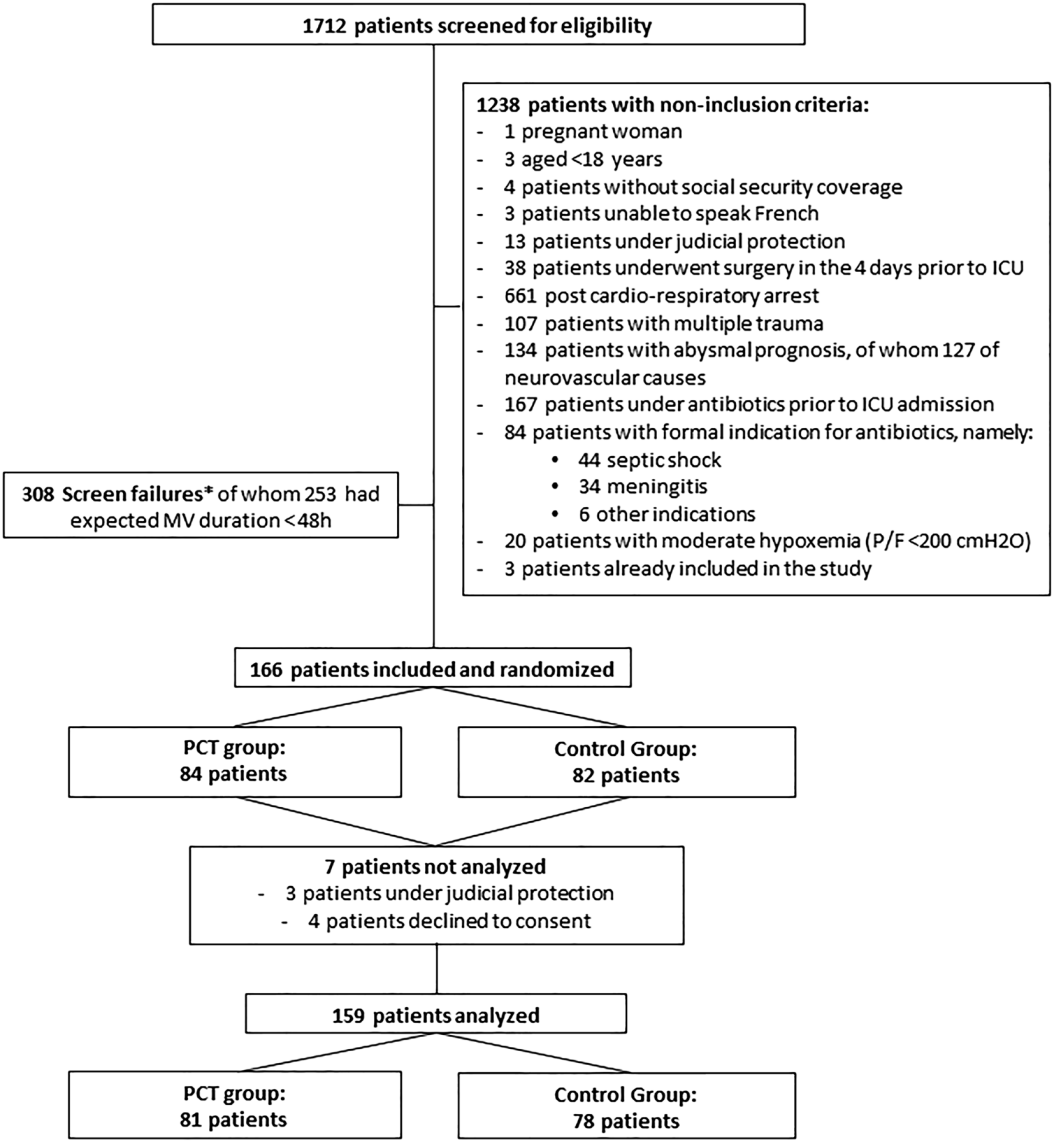
Flowchart of the study population. *Screen failures = patients not included in the study for undocumented reasons

From 24/2/2015 to 28/8/2019, in the five participating centers, a total of 1712 patients were intubated for coma, of whom 166 were included in the study. The flowchart of the study population is shown in Fig. 3. Data from seven randomized patients could not be used: three patients were under legal protection at the time of inclusion, and four patients refused their consent after regaining capacity. A final total of 159 patients were thus included in the final intention-to-treat analysis, 81 and 78 in the PCT group and the control group respectively. The characteristics of the study population are shown in Table 1. Average SAPS II score within 24 h after ICU admission was significantly higher in the PCT group (48.9 ± 10.9) than in the control group (45.2 ± 9.5, *p* = 0.024). There was no other significant difference between groups.

**Table 1:** Baseline characteristics of the study population, by group

	PCT group (*n* = 81)	Control group (*n* = 78)	*P*
Age (years)	53.8 ± 17.8	52.0 ± 17.2	0.54
Male sex	53 (65)	41 (53)	0.1
Origin before ICU admission			0.68
Home	69 (86)	69 (89)	
Medical ward	11 (14)	9 (12)	
Comorbidities			
Smoking	35 (43)	32 (41)	0.78
Chronic alcohol consumption	28 (35)	20 (26)	0.22
COPD/asthma	3 (4)	3 (4)	1
Chronic heart failure	5 (6)	2 (3)	0.44
Chronic renal failure	2 (3)	2 (3)	1
Chronic respiratory failure	1 (1)	0	1
SOFA Score	6.2 ± 1.9	6.1 ± 1.9	0.76
SAPS II Score	48.9 ± 10.9	45.2 ± 9.5	0.02
Glasgow Score before intubation	4 [3, 6]	5 [3, 6]	0.47
PaO_2_/FiO_2_ ratio at inclusion	308.4 ± 105.6	317.0 ± 94.8	0.59
Coma etiology			
Toxic	39 (48)	41 (53)	0.57
Epilepsy	36 (44)	29 (37)	0.35
Stroke	17 (10)	11 (6)	0.25
Metabolic disorders	4 (5)	1 (1)	0.37
Cerebral tumor	1 (1)	4 (4)	0.2
Hemodynamic	0	5 (6)	0.03
Other	3 (4)	4 (8)	0.72

*PCT* procalcitonin, *COPD* chronic obstructive pulmonary disease, *SOFA* Sequential Organ Failure Assessment, *SAPS II* Simplified Acute Physiological Score II, *IQR* interquartile range

**Fig. 4:**
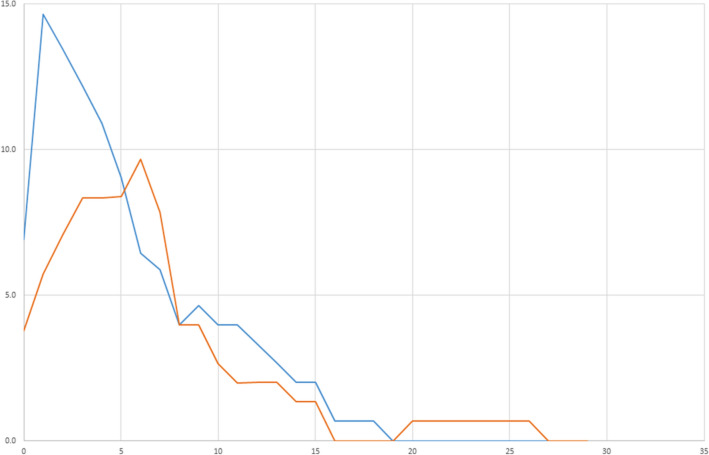
Proportion of patients with antibiotics initiated over time in the ICU (days since admission to ICU). Orange line = control group; blue line = PCT group. ICU, intensive care unit; MV, mechanical ventilation; PCT, procalcitonin

The details of infection and antibiotic use are given in Table 2. The proportion of patients in whom antibiotics were initiated during the ICU stay is displayed in Fig. 4. During the ICU stay, when antibiotics were used, the most frequent site of infection was the lung. A total of 67 patients (43%) received antibiotics during their ICU stay, with no significant difference between groups [37 patients (46%) in the PCT group versus 30 patients (40%) in the control group (*p* = 0.43)]*.* The average duration of antibiotic therapy at 7 and 15 days after admission to the ICU were respectively 1.9 ± 2.4 and 2.7 ± 3.8 days, and there was no significant difference between groups. There was no significant difference in the number of antibiotic-free days between groups (Table 2). The number of patients with antibiotics initiated beyond 3 days after inclusion was 9 (11.1%) in the PCT group, and 8 (10.3%) in the control group (*p* = 0.86). There was no significant difference in the number of patients still under antibiotic therapy at discharge from the ICU [10 patients (12.3%) in the PCT group, vs 10 (12.8%) in the control group, *p* = 0.93)].

**Table 2:** Details of infections and antibiotic prescriptions

	PCT Group (*n* = 81)	Control group (*n* = 78)	*p*	Adjusted p*
Suspected site of infection (at 7 days)				
Lungs	30 (37)	25 (32)	0.51	0.68
Urinary tract	2 (3)	4 (5)	0.44	0.26
Digestive tract	1 (1)	0	1	0.95
Catheter	1 (1)	0	1	0.95
Other	6 (7)	3 (4)	0.5	0.53
Severity criteria				
Severe sepsis	5 (7)	7 (13)	0.3	0.23
Septic shock	4 (6)	3 (5)	1	0.72
No. exposed to antibiotics	37 (46)	30 (40)	0.43	0.61
Timing between admission to ICU and initiation of antibiotics (days)	1.3 ± 2.4	1.5 ± 2.9	0.74	0.72
No. of days on antibiotics during first week	2.1 ± 2.5	1.7 ± 2.3	0.31	0.37
No. of days on antibiotics during first 15 days	3.0 ± 4.1	2.3 ± 3.4	0.31	0.41
Antibiotic consumption in total DDD in ICU	11.5 ± 22.4	8.0 ± 14.7	0.3	0.48
No. of antibiotic-free days up to 15 days	13 ± 2.7	13.5 ± 2.9	0.30	0.38

*PCT* procalcitonin, *No*. number, *DDD* Defined Daily Doses

^*^Adjusted for SAPS II score

In the PCT group, the decisional algorithm for initiation of antibiotic therapy was followed in 68 patients (84%). In 13 patients (16%), this algorithm was not followed: 8 patients (73%) received antibiotics despite PCT < 0.5 µg/L, due to criteria of clinical severity (septic shock or hypoxia). In 5 patients, no reasons for non-adherence with the algorithm could be identified: 1 patient did not receive antibiotics despite a PCT value of 0.56 µg/L; 1 patient received antibiotics despite the absence of any PCT measurement; and 3 patients received antibiotics despite PCT levels < 0.5 µg/L.

The microbes responsible for infection are described in Additional file 1: Table S1. No anaerobic microbe was identified in respiratory samples. Sixteen respiratory infections were polymicrobial.

The comparison between exposure to antibiotics and the results of the bacteriological cultures is described in Additional file 1: Table S2. During follow-up in the ICU, 3 patients had recurrent infection (2 in the PCT group, and 1 in the control group) and one superinfection occurred in the control group. No pulmonary abscess, pleurisy or *Clostridium difficile* infection was observed. Systematic searching for multistrain-resistant bacterial did not show any significant difference between groups [2 patients (3%) in the PCT group and 2 patients (3%) in the control group, *p* = 1.0].

Patient outcomes and life support therapies are detailed in Table 3. There was no significant difference in SOFA score between groups up to day 7. No comparison could be performed thereafter due to the low number of patients still hospitalized. There was no significant difference between groups in the use of life support therapies (catecholamines or renal replacement therapy). The average number of days on MV was significantly higher in the PCT group (3.7 ± 3.6 days) than in the control group (2.7 ± 2.5 days) (*p* = 0.03). The length of ICU stay was also longer in the PCT group (6.4 ± 6.5 vs 4.6 ± 3.5 in the control group, *p* = 0.04). After adjustment for SAPS II score, the difference in length of stay and duration of mechanical ventilation between groups was no longer significant. Total length of hospital stay was similar between groups. Seventeen patients (11.3%) died during the 3-month follow-up, with no significant difference between groups.

**Table 3:** Life support therapies and outcomes

	PCT group (*n* = 81)	Control group (*n* = 78)	*p*	Adjusted p*
SOFA Score				
Day 2	4.3 ± 3.0	3.6 ± 3.3	0.26	0.94
Day 3	4.3 ± 2.6	4.1 ± 3.4	0.72	0.93
Day 5	3.9 ± 2.9	4.4 ± 2.7	0.57	0.84
Day 7	3.8 ± 2.9	3.1 ± 2.8	0.46	0.69
No. of days on mechanical ventilation	3.7 ± 3.6	2.7 ± 2.5	0.03	0.07
Catecholamines	28 (35)	18 (23)	0.11	0.31
Renal replacement therapy	3 (4)	0	0.25	0.95
Length of ICU stay (days)	6.4 ± 6.5	4.6 ± 3.5	0.04	0.06
Death in ICU	5 (6.1)	4 (5.1)	0.78	0.80
Death in-hospital (after ICU discharge)	3 (3.7)	4 (5.1)	0.72	0.38
Death at 28 days	5 (6.2)	9 (11.5)	0.27	0.20
Death at 90 days	8 (9.9)	9 (12.8)	0.73	0.42

*SOFA* Sequential Organ Failure Assessment, *ICU* intensive care unit

^*^Adjusted for SAPS II score

## Discussion

This study shows that the use of a decisional algorithm based on PCT values to guide the introduction of antibiotic therapy in ICU patients intubated for coma did not reduce the duration of exposure to antibiotics.

In our study, there was no significant difference in the duration of exposure to antibiotics between the two groups. The proportion of patients in the control group who received antibiotics was 40%, which is in line with the study by Lascarrou et al. [28], in which 39.2% of patients were treated with antibiotics. In that study, antibiotic therapy was initiated in case of suspected inhalation pneumonia, with clinical suspicion based on clinical, biological and radiological criteria similar to those used in our control group.

The average number of days spent on MV and the length of ICU stay were longer in the PCT group in our study. These differences may be related to numerous factors, notably a more severe profile of disease in the patients in the PCT group, reflected by the higher SAPS II score in this group [48.9 ± 10.9 in the PCT group versus 45.2 ± 9.5 in the control group, *p* = 0.024)]. Indeed, after adjustment of the comparison for SAPS II score, the difference no longer reached statistical significance. The mortality rate at 3 months was 11.3%, albeit without any significant difference between groups. This rate is lower than other reports in the literature for non-trauma coma patients admitted to ICU (25–87%) [33]. The lower mortality in our population may be explained by different etiologies. Indeed, there were more coma patients with toxic etiology in our study (48% in the PCT group and 53% in the control group, *p* = *0.57*) than in other studies reported in the literature (< 1–39%), while the proportion of neurovascular disease (10% in the PCT group and 17% in the control group, *p* = *0.25*) was lower than reported elsewhere (6–54%) [33]. Finally, patients with coma secondary to cardiac arrest, responsible for the majority of post-anoxic comas, were not included in our study. Since mortality is reportedly lower in coma of toxic origin (0–39%) compared to other etiologies (neurovascular 60–95% and post-anoxic 54–89%), the distribution of disease etiologies in our population may have contributed to the lower overall mortality [33].

The different etiological profile observed in our study could be explained by the non-inclusion criteria applied. Firstly, moribund patients whose prognosis was judged to be poor were not included. Among this type of patient, there is a high proportion of patients with coma from neurovascular causes (134 patients were excluded for this reason, of whom 127 had coma of neurovascular origin). Secondly, patients with coma after cardiac arrest were also ineligible, based on the fact that PCT levels are naturally higher after cardiac arrest, but without any relation with infection, particularly early post-intubation pneumonia [34, 35].

For the decisional algorithm used in this study to guide introduction of antibiotic therapy, we chose a PCT threshold of 0.5 µg/L. This cut-off was chosen based on the prospective, observational study by Pusch et al. [26], in which the study population comprised patients with coma (defined as a Glasgow score ≤ 8) further to closed head injury. In their study, PCT values were assessed at 12, 24, 36 and 72 h after the trauma, and were found to be significantly lower in patients without bronchoscopic or radiological signs of aspiration of gastric content. Indeed, in this group, PCT values did not exceed 0.418 µg/L.

The second algorithm, used to guide discontinuation of antibiotic therapy, was similar to that used in the study by De Jong et al. [24]. In their prospective, multicenter, randomized, controlled, open-label study investigating the efficacy and safety of procalcitonin guidance in reducing the duration of antibiotic treatment, De Jong et al. included 1575 patients. They observed a reduction in the duration of treatment and the number of daily defined doses in critically ill patients with a presumed bacterial infection, associated with a significant decrease in mortality in the PCT group. This remains the largest study published to date on this topic in ICU patients, and the algorithm used is the most robust and comprehensive one available in the literature, to guide antibiotic therapy based on PCT in the ICU setting.

### Study strengths and limitations

Our study has several strengths. Firstly, it was a multicenter study. Second, the discontinuation algorithm used here has previously been validated in a large study. Third, there was a high rate of compliance with the decisional algorithm for initiation of antibiotic therapy in our study (84%). Our study also has some limitations. Firstly, although it was a multicenter study, there were only 5 centers, with an open-label design. Second, some patients in the PCT group may have been discharged from the ICU before the discontinuation algorithm could be implemented if they were still under antibiotic therapy with PCT > 0.5 µg/L at the time of discharge. The study aim was to assess the duration of antibiotic therapy in the ICU, and the continued surveillance of PCT and antibiotic use after ICU discharge was not logistically possible. Third, no anaerobic bacteria were identified in respiratory samples. The isolation of anaerobic microbes is difficult due to the need for specific sampling techniques, transport modalities and culture medium. The failure to identify any anaerobic bacteria may be due to a failure to comply with the optimal conditions for sampling and culture of these microbes. Nevertheless, this absence of anaerobic microbes is congruent with the changing distribution of microbe types, particularly the reduction in the proportion of anaerobic bacteria observed in inhalation pneumonia over the last 40 years [5, 28, 36, 37]. Finally, in our study, the average duration of exposure to antibiotics during the first 15 days was 3.0 ± 4.1 days in the PCT group, and 2.3 ± 3.4 days in the control group. This is shorter than the mean duration used to calculate the sample size (6.2 ± 3.3 days), and therefore, the study may be underpowered to show an effect. Nevertheless, the shorter duration was observed in both groups, and management was the same in both groups, suggesting the absence of any influence of PCT on the management strategy.

## Conclusion

In conclusion, the use of PCT assessment to guide the introduction and discontinuation of antibiotic therapy did not significantly reduce exposure to, or duration of antibiotics in ICU patients intubated for coma. These findings warrant confirmation in larger prospective studies.

## Supplementary Information

**Additional file 1:** **Table S1.** Microbes responsible for infection, by type of sample and by randomization group. **Table S2.** Comparison between exposure to antibiotics and results of bacteriological cultures.
