# Supplementary material for: Impact on antimicrobial consumption of procalcitonin-guided antibiotic therapy for pneumonia/pneumonitis associated with aspiration in comatose mechanically ventilated patients: a multicenter, randomized controlled study
Source: Ann Intensive Care. 2021 Oct 12;11:145. doi: 10.1186/s13613-021-00931-4 (PMC8505789; doi:10.1186/s13613-021-00931-4)
Supplement: Supplementary file 1 — Additional file 1: Table S1. Microbes responsible for infection, by type of sample and by randomization group. Table S2. Comparison between exposure to antibiotics and results of bacteriological cultures. [file 13613_2021_931_MOESM1_ESM.docx]

**Impact on antimicrobial consumption of procalcitonin-guided antibiotic therapy for Pneumonia / pneumonitis Associated with aspiration in Comatose Mechanically Ventilated patients**: **a multicenter, randomized controlled study.**

**Supplementary Material**

**Table of Contents:**

**Supplementary Table S1:** Microbes responsible for infection, by type of sample and by randomization group

**Supplementary Table S2:** Comparison between exposure to antibiotics and results of bacteriological cultures

**Supplementary Table S1:** **Isolated microbes, by type of sample and by randomization group***

|  | **Tracheal aspiration and BAL** | | **Blood culture** | | **Urine culture** | | **Other** | |
| --- | --- | --- | --- | --- | --- | --- | --- | --- |
|  | **PCT** | **Control** | **PCT** | **Control** | **PCT** | **Control** | **PCT** | **Control** |
| **GRAM NEGATIVE** | **22** | **23** | **10** | **7** | **3** | **3** | **3** | **2** |
| **Enterobacteria** | **15** | **19** | **8** | **4** | **2** | **2** | **2** | **2** |
| *Escherichia coli* | *4* | *13* | *3* | *3* | *2* | *1* | *2* | *0* |
| *Citrobacter pilori* | *0* | *1* | *0* | *0* | *0* | *0* | *0* | *0* |
| *Citrobacter krusei* | *2* | *2* | *1* | *0* | *0* | *0* | *0* | *0* |
| *Serratia liquifaciens* | *1* | *1* | *0* | *0* | *0* | *0* | *0* | *0* |
| *Serratia marcescens* | *0* | *0* | *0* | *0* | *0* | *1* | *0* | *0* |
| *Klebsiella oxytoca* | *1* | *1* | *1* | *0* | *0* | *0* | *0* | *0* |
| *Klebsiella pneumonia* | *1* | *0* | *0* | *1* | *0* | *0* | *0* | *0* |
| *Enterobacter cloacae* | *0* | *0* | *0* | *0* | *0* | *0* | *0* | *1* |
| *Enterobacter aerogenes* | *1* | *0* | *0* | *0* | *0* | *0* | *0* | *0* |
| *Proteus mirabilis* | *2* | *1* | *3* | *0* | *0* | *0* | *0* | *0* |
| *Proteus vulgaris* | *1* | *0* | *0* | *0* | *0* | *0* | *0* | *1* |
| *Morganella morganii* | *2* | *0* | *0* | *0* | *0* | *0* | *0* | *0* |
| **Pseudomonas aeruginosa** | **3** | **0** | **0** | **2** | **0** | **0** | **1** | **0** |
| **Stenotrophomonas maltophilia** | **0** | **0** | **0** | **1** | **0** | **0** | **0** | **0** |
| **Haemophilus influenzae** | **2** | **4** | **1** | **0** | **1** | **1** | **0** | **0** |
| **Branhamella catarrhalis** | **2** | **0** | **1** | **0** | **0** | **0** | **0** | **0** |
|  |  |  |  |  |  |  |  |  |
|  |  |  |  |  |  |  |  |  |
| **GRAM POSITIVE** | **27** | **34** | **21** | **11** | **8** | **5** | **10** | **3** |
| **Staphylococcus aureus** | **12** | **19** | **13** | **4** | **2** | **2** | **3** | **2** |
| **Staphylocoque coagulase negative** | **6** | **6** | **3** | **4** | **1** | **1** | **0** | **1** |
| **Streptococcus pneumoniae** | **4** | **6** | **1** | **2** | **2** | **2** | **3** | **0** |
| **Streptococcus (other)** | **1** | **1** | **2** | **0** | **1** | **0** | **1** | **0** |
| *Streptococcus agalactiae* | *0* | *0* | *0* | *0* | *1* | *0* | *0* | *0* |
| *Streptococcus anginosus* | *0* | *1* | *0* | *0* | *0* | *0* | *1* | *0* |
| *Streptococcus gallolyticus* | *0* | *0* | *2* | *0* | *0* | *0* | *0* | *0* |
| *Stresptococcus mitis* | *1* | *0* | *0* | *0* | *0* | *0* | *0* | *0* |
| **Enterococcus faecalis** | **3** | **2** | **1** | **1** | **1** | **0** | **1** | **0** |
| **Actinomyces odontolyticus** | **0** | **0** | **0** | **0** | **1** | **0** | **0** | **0** |
| **Pasteurella multocida** | **1** | **0** | **0** | **0** | **0** | **0** | **0** | **0** |
| **Anaerobic** | **0** | **0** | **1** | **0** | **0** | **0** | **2** | **0** |
| *Clostridium perfringens* | *0* | *0* | *0* | *0* | *0* | *0* | *2* | *0* |
| *Lactobacillus* | *0* | *0* | *1* | *0* | *0* | *0* | *0* | *0* |

PCT, procalcitonin group ; BAL, bronchoalveolar lavage

* Totals exceed the number of patients, since patients could have more than one sample, and from more than one site.

**Supplementary Table S2: Comparison between exposure to antibiotics and results of bacteriological cultures**

For the whole study population (N=159):

|  | No antibiotics | Antibiotics |
| --- | --- | --- |
| Bacteriology negative | 58 (84.1) | 11 (15.9) |
| Bacteriology positive | 34 (37.8) | 56 (62.2) |

In the Procalcitonin-guided group (N=81):

|  | No antibiotics | Antibiotics |
| --- | --- | --- |
| Bacteriology negative | 27 (84.4) | 5 (15.6) |
| Bacteriology positive | 17 (34.7) | 32 (65.3) |
